# Knee hyperextension is not associated with anterior knee laxity, subjective knee function or revision surgery after anterior cruciate ligament reconstruction in children and adolescents

**DOI:** 10.1002/ksa.12707

**Published:** 2025-05-26

**Authors:** Frida Hansson, Anders Stålman, Gunnar Edman, Per‐Mats Janarv, Eva Bengtsson Moström, Riccardo Cristiani

**Affiliations:** ^1^ Department of Molecular Medicine and Surgery, Section of Sports Medicine Karolinska Institutet Stockholm Sweden; ^2^ Stockholm Sports Trauma Research Center (SSTRC), FIFA Medical Centre of Excellence Stockholm Sweden

**Keywords:** ACL reconstruction, adolescents, children, knee hyperextension, laxity, revision

## Abstract

**Purpose:**

To evaluate whether contralateral knee hyperextension (KHE) is associated with anterior knee laxity, subjective knee function or revision surgery after primary anterior cruciate ligament reconstruction (ACLR) in patients <18 years.

**Methods:**

Patients <18 years who underwent primary ACLR at Capio Artro Clinic, Stockholm, Sweden between January 2002 and March 2017 were identified. They were dichotomised into a ‘hyperextension’ group (≤−5°) and ‘no hyperextension’ group (>−5°) depending on preoperative contralateral passive knee extension degree. Anterior knee laxity (KT‐1000 arthrometer) was measured preoperatively and 6 months post‐operatively. The knee injury and osteoarthritis outcome score (KOOS) was collected preoperatively and after 2 years. Revision ACLR within 5 years after primary ACLR was captured in the Swedish National Knee Ligament Registry.

**Results:**

1250 patients (63.6% female [*n* = 795]; mean age 15.5 ± 1.5 years) were included (hyperextension group: 52.9% [*n* = 661]). Mean extension was −6.1 ± 2.2° in the hyperextension group and 0 ± 0.7° in the no hyperextension group. Hamstring autograft was used in 93.3% (1166 out of 1250). No significant difference between the groups was seen in anterior knee laxity or in the rate of surgical failure at 6 months post‐operatively (side‐to‐side difference: >5 mm) (hyperextension group, 6.6% [32 out of 484 patients] vs. no hyperextension group, 6.8% [29 out of 428 patients]; *p* = ns). Statistically significant but non‐clinically relevant intergroup differences were seen in the KOOS Sport/Recreation and Quality of Life subscales after 2 years. The rate of revision ACLR within 5 years was 11.1% (119 out of 1073 patients). The hazard for revision ACLR in the hyperextension group was not significantly different from the no hyperextension group (hazard ratio, 0.91; 95% confidence interval, 0.63–1.31; *p* = ns).

**Conclusions:**

There was no significant association between preoperative passive contralateral KHE and anterior knee laxity, subjective knee function or the risk of revision ACL surgery in paediatric patients. These findings suggest that KHE alone should not preclude the use of hamstring tendon grafts in children and adolescents undergoing ACL reconstruction. The study found a high rate of revision ACL surgery in this paediatric population.

**Level of Evidence:**

Level III.

**Abbreviations**
ACL: anterior cruciate ligament
ACLR: anterior cruciate ligament reconstruction
ALL: anterolateral ligament
BPTB: bone‐patellar tendon‐bone
GJH: generalised joint hypermobility
HT: hamstring tendon
IKDC: International Knee Documentation Committee
KHE: knee hyperextension
LM: lateral meniscus
LSI: limb symmetry index
MM: medial meniscus
ROM: range of motion
SLH: single‐leg hop
SNKLR: Swedish National Knee Ligament Registry
STS: side‐to‐side
WB: weight bearing

## INTRODUCTION

An anterior cruciate ligament (ACL) tear is one of the most common knee injuries among children and adolescents, with an increasing incidence reported over recent decades [6, 49]. For patients who report instability and/or wish to return to pivoting sports, an ACL reconstruction (ACLR) is generally recommended [3, 15]. In children and adolescents, the risk for graft failure and revision ACLR has been reported to be consistently higher than in adults [25, 48, 50].

Several factors may affect outcomes following ACLR in younger populations. One such factor is generalised joint hypermobility (GJH), which is associated with inferior outcomes after ACLR in adults. A recent review by Sundemo et al. [46] reported greater post‐operative knee laxity and poorer patient‐reported outcomes in adults with GJH. Knee hyperextension (KHE) is a specific component of GJH [4] and is highly prevalent in individuals with GJH. KHE has been identified as a risk factor for ACL injury in both adults [41] and female adolescents [38]. Biomechanical cadaveric studies have demonstrated that passive KHE places a greater force on the ACL and can lead to impingement [27, 29]. These biomechanical findings suggest that KHE could also have a clinical impact on ACLR outcomes, which has been shown in previous studies in adults [20, 23, 31, 32, 47].

In adults, a few clinical studies have investigated the relationship between KHE and postoperative knee laxity, yielding conflicting results. Some studies have reported a significant association between KHE and increased anterior tibial translation following ACLR [23, 32, 47]. However, when compared with the contralateral, uninjured knee, Sundemo et al. [47] found no difference in side‐to‐side (STS) measurements preoperatively or at 6 months postoperatively. Additionally, KHE has been linked to inferior subjective knee function and higher graft failure rates when using hamstring tendon (HT) autografts [20, 23, 31]. Conversely, other studies found no clinically relevant association between KHE and inferior outcomes. For instance, Benner et al. [5] and Edman et al. [18] reported no significant relationship between KHE and subjective knee function or revision rates in patients undergoing ACLR with bone‐patellar tendon‐bone (BPTB) or HT grafts, respectively. Furthermore, Cristiani et al. [9] found no increased risk of revision surgery within 5 years after ACLR in patients younger than 20 years with KHE. Despite the growing body of research in adults, there is limited evidence regarding the effects of KHE on ACLR outcomes in paediatric and adolescent populations. This gap underscores the need for further studies to explore whether the adult findings can be applied to younger individuals. It is suggested that in patients with joint hypermobility, alternative graft choices, such as BPTB grafts, might be more suitable than HT autografts [20]. In addition, it has also been suggested that an anterolateral ligament (ALL) reconstruction should be considered when performing ACLR with HT autografts in patients with ligamentous hyperlaxity in order to reduce the risk of failure and improve stability [24].

The purpose of this study was to evaluate whether physiologic passive contralateral KHE is associated with anterior knee laxity, subjective knee function, or revision surgery after primary ACLR in patients younger than 18 years. We hypothesised that passive contralateral KHE (≤−5°) was associated with increased anterior knee laxity, inferior subjective knee function, and higher rates of revision ACLR in comparison with no KHE (>−5°).

## MATERIALS AND METHODS

Ethical permission for this study was obtained from the Regional Ethics Committee, Karolinska Institutet (Diarie number: 2016/1613‐31/2).

**Figure 1:**
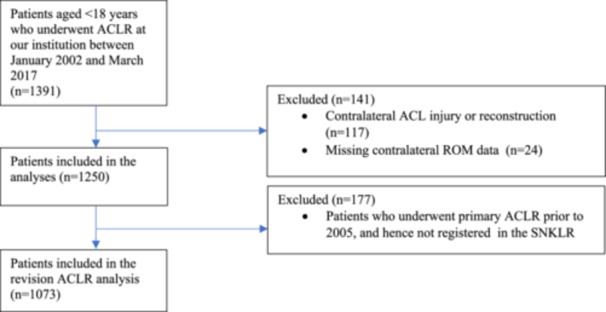
Flowchart of the included patients. ACLR, anterior cruciate ligament reconstruction; n, number of patients; ROM, range of motion; SNKLR, Swedish National Knee Ligament Registry.

Patients younger than 18 years who underwent primary ACLR with either BPTB or HT autografts at Capio Artro Clinic, Stockholm, Sweden between January 2002 and March 2017 were assessed for eligibility. Patients with contralateral ACLR or no data regarding preoperative contralateral knee range of motion (ROM) were excluded (Figure 1).

### Surgical technique and rehabilitation

All ACLRs were performed using either an autologous single‐bundle HT or BPTB technique. The HT grafts were primarily prepared from the semitendinosus tendon, with the gracilis tendon harvested if the semitendinosus graft diameter was <7 mm. BPTB grafts were harvested with two bone blocks and the central third of the patellar tendon. No BPTB were used in skeletally immature patients. The femoral tunnels were drilled using either a transtibial or anteromedial portal technique, and transphyseal drilling was used for both the femoral and tibial tunnels in patients with open physes. Femoral fixation was routinely performed using an Endobutton (Smith & Nephew) or TightRope (Arthrex) fixation device. Tibial fixation was achieved with an AO bicortical screw (Smith & Nephew) and a washer as a post, or with an interference screw. No interference screws were used in patients with open physes. Tibial fixation was performed after the femoral fixation, at about 90° of flexion for HT grafts and at 20 degrees of flexion for BPTB grafts. There was no difference in fixation angle between the hyperextension and the no hyperextension group. None of the patients included in the study underwent a lateral extra‐articular procedure, such as ALL reconstruction or lateral extra‐articular tenodesis.

The meniscal tears were repaired arthroscopically. For tears in the posterior horn and body of the meniscus, an all‐inside technique with Fast‐Fix suture devices (Smith & Nephew) was used. An outside‐in technique with No. 0 PDS sutures (Ethicon) was used for anterior tears. The post‐operative rehabilitation followed a standardised protocol. For patients aged ≥15 years, full weight bearing (WB) and full ROM were allowed as tolerated. In cases of meniscal repair, a hinged knee brace was used for 6 weeks post‐operatively with ROM progression as follows: Weeks 1 and 2: 0°–30°, Weeks 3 and 4: 0°–60° and Weeks 5 and 6: 0°–90° flexion. For patients under the age of 15 years, post‐operative protocols varied. At the beginning of the study period (2002–2011), they followed the same protocol as older patients. Since 2012, limited WB and ROM were recommended, with partial WB and a brace locked at 30° flexion for 3 weeks, followed by full WB and ROM limits of 10°–90° for another 3 weeks [21]. The purpose of the ROM limits was to minimise ACL tension at 30° flexion [29, 30].

Quadriceps strengthening was restricted to closed kinetic chain exercises during the first 3 months. Return to sports was individualised and required a limb symmetry index (LSI) of ≥90% on isokinetic quadriceps and hamstring strength, as well as on the single‐leg hop (SLH) test for distance. Return to sport was not allowed earlier than 6 months post‐operatively [11, 12].

### ROM measurements

Passive ROM was measured bilaterally by using a goniometer. All ROM measurements were performed preoperatively. The patient was examined supine with the heels on a bolster. The goniometer was centred on the lateral femoral condyle. The greater trochanter was used as a reference for alignment of the lateral midline of the femur, and the lateral malleolus was used as a reference for alignment of the lateral midline of the fibula [39]. Experienced sports medicine physical therapists performed all the measurements at our outpatient clinic. In this study, the KHE of the uninjured knee was used to avoid eventual effects on ROM measurements due to the ACL injury. Depending on the degree of passive knee extension, the cohort was dichotomised into two groups: ‘hyperextension’ (≤−5°) and ‘no hyperextension’ (>−5°).

### Muscle strength and SLH test performance assessment

Isokinetic concentric extension and flexion strengths were measured 6 months post‐operatively. The Biodex System 3 (Biodex Medical Systems) was used. After a 10‐min standardised warm‐up, the test was performed with a ROM between 90° and 10° of knee flexion, starting with the uninjured knee. Two to three practical trials were allowed before the test. Each patient performed five maximum quadriceps and hamstring contractions with each leg. The highest achieved values of the quadriceps and hamstring torques were registered.

The SLH test for distance was used to assess functional hop performance. The patient stood on one leg and was instructed to jump straight ahead as far as possible, and land stable on the same leg. The hop was repeated in cases where the landing was unstable. The patients were allowed to perform several practical trials until they felt confident about the test. The best trial out of three was registered, and the test always started with the contralateral uninjured knee.

### Laxity

Preoperative and 6‐month post‐operative anterior knee laxity was measured by experienced physiotherapists at our outpatient clinic using a KT‐1000 arthrometer (MEDmetric Corp.) [51]. The median value of at least three repeated measurements for each knee was registered. Measurements were taken at 20° knee flexion with a 134‐N anterior tibial load. The mean pre‐ and postoperative STS differences between operated and non‐operated knees were calculated. The differences were expressed in millimetres. STS laxity was also stratified into three groups (≤2, 3–5 and >5 mm) according to the International Knee Documentation Committee (IKDC) examination form [22].

### Patient‐reported outcome measures (PROMs)

Subjective knee function was evaluated preoperatively and at 2 years post‐operatively using the Knee Injury and Osteoarthritis Outcome Score (KOOS) [43].

### Revision ACLR

Patients who underwent revision ACLR at our institution or other institutions in the country were identified through their unique Swedish personal identity number [34] in the Swedish National Knee Ligament Registry (SNKLR) [26]. Patients with primary ACLR prior to 2005 were excluded from the revision ACLR analysis due to missing data in the SNKLR, which started registration of ACLRs in 2005. The follow‐up time from the primary ACLR was 5 years.

### Data sources

Demographic data (age and sex), time from injury to surgery, pre‐injury Tegner activity level, graft type, presence of cartilage injury, meniscus injury (medial or lateral), meniscus surgery (classified as resection or repair of the medial meniscus [MM] or lateral meniscus [LM]), 6‐month isokinetic (extension and flexion) strength, and SLH test performance were collected from our local database. Preoperative and post‐operative KT1000 arthrometric laxity measurements were also collected from our local database. The KOOS subscale scores were collected from the SNKLR.

### Statistical methods

The computations of descriptive statistics and statistical analyses were performed using SPSS software (﻿version 27.0, IBM Corp.). All variables were summarised using standard descriptive statistics, such as mean, standard deviation (SD) and frequency. All distributions were checked for severe deviations from the normal distribution. Parametric statistics were preferred for the analysis of approximately normally distributed variables. Independent‐samples Student's *t* test was used for the comparison of continuous variables, whereas the Pearson chi‐square test was used for the comparison of categorical variables.

Differences in preoperative STS laxity between the injured and non‐injured knees were analysed using analysis of covariance (ANCOVA) with sex and time from injury to ACLR as covariates [10]. For post‐operative STS difference, MM resection was also included as a covariate [10, 13]. The stratified pre‐ and post‐operative STS laxity according to the IKDC examination form [22] was analysed using the Pearson chi‐square test for trends. A sub‐analysis of mean pre‐ and post‐operative STS difference in the subgroups knee extension ≤−10°, −5° to −9°, −1° to −4° and >0° was performed with one‐way analysis of variance.

Preoperative KOOS subscale scores were analysed using an ANCOVA, with sex as a covariate. Two‐year post‐operative KOOS subscale scores were analysed using an ANCOVA, with sex, time from injury to surgery, and medial meniscal resection as covariates. These potential confounders were chosen as they differed between the groups, and a potential association with subjective outcomes has been previously reported [10, 13]. To compare the hazard of revision ACLR within 5 years after the primary surgery between the KHE (≤−5°) and no KHE (>−5°) groups, a Cox regression analysis was used. The covariates were sex, time from injury to surgery and medial meniscal resection. These potential confounders were selected due to the significant differences between the two groups, and since these variables have been shown to be associated with revision ACLR in previous studies [8]. The results of the Cox regression analysis were reported as hazard ratios with 95% confidence intervals (CIs). The significance level in all analyses was 5% (two‐tailed).

## RESULTS

A total of 1391 patients younger than 18 years were available in our database and eligible for inclusion. After excluding patients with no available contralateral ROM measurements (n = 24) and patients with a contralateral ACL injury or reconstruction (*n* = 117), 1250 patients were included (Figure 1). The mean age for the entire cohort was 15.5 ± 1.5 years. A total of 661 patients (52.9%) had a contralateral passive knee extension ≤−5° and constituted the hyperextension group. The mean knee extension in the hyperextension group was −6.1 ± 2.2° and in the no hyperextension group 0 ± 0.7°. Demographic and clinical data for the two groups, ‘hyperextension’ (≤−5°) and ‘no hyperextension’ (>−5°) groups, are presented in Table 1. In patients younger than 15 years (272 patients), 154 (56.7%) had a contralateral passive knee extension ≤−5°.

**Table 1:** Patient characteristics.^a^

Variable		Hyperextension (≤−5°) (*n* = 661)	No hyperextension (>−5°) (*n* = 589)	*p*
*Preoperative factors*				
Knee extension in degrees	M ± SD (range)	−6.1 ± 2.2 (−20 to −5)	0 ± 0.7 (−4 to 5)	<0.001
Age at surgery, years	M ± SD	15.4 ± 1.5	15.5 ± 1.5	ns
Sex				
Female		446 (67.5)	349 (59.3)	0.003
Male		215 (32.5)	240 (40.7)	
Time from injury to surgery, months	M ± SD	8.9 ± 10.1	8.0 ± 11.6	0.038
Patients with available data		*n* = 610	*n* = 543	
Pre‐injury Tegner activity level	Median (range)	8 (1–10)	8 (2–10)	
High, >5		519 (92.8)	454 (92.3)	ns
Low, ≤5		40 (7.2)	38 (7.7)	
Patients with available data		*n* = 559	*n* = 492	
*Intra‐operative factors*				
Graft type				
BPTB		50 (7.6)	34 (5.8)	ns
HT		611 (92.4)	555 (94.2)	
Graft diameter (for HT graft)		8.3 ± 0.8	8.3 ± 0.8	ns
Medial meniscus injury		151 (22.8)	118 (20.0)	ns
Lateral meniscus injury		196 (30.0)	152 (25.8)	ns
Medial meniscus surgery				
Resection		60 (9.1)	31 (5.3)	0.010
Repair		67 (10.1)	66 (11.2)	ns
Lateral meniscus surgery				
Resection		105 (15.9)	77 (13.1)	ns
Repair		59 (8.9)	56 (9.5)	ns
Cartilage injury				
Yes		55 (8.3)	39 (6.6)	ns
No		606 (91.7)	550 (93.4)	
*Post‐operative factors (6 months)*				
Isokinetic quadriceps strength LSI	M ± SD	89.7 ± 14.5	90.9 ± 13.2	ns
Patients with available data		*n* = 606	*n* = 529	
Isokinetic hamstring strength LSI	M ± SD	90.4 ± 16.2	93.1 ± 17.5	0.004
Patients with available data		*n* = 605	*n* = 528	
Single leg hop test LSI	M ± SD	95.6 ± 10.9	96.7 ± 9.1	ns
Patients with available data		*n* = 558	*n* = 481	

*Note*: *M* ± SD: mean ± standard deviation.

Abbreviations: BPTB, bone‐patellar‐tendon‐bone; HT, hamstring tendon; LSI, limb symmetry index; n, number; ns, non‐significant; SD, standard deviation.

^a^ Data are reported as *n* (%) unless otherwise indicated.

### Laxity

There were no significant differences in the preoperative or post‐operative mean STS laxity or in the STS laxity values, stratified according to the IKDC examination form [22], between the two groups (Table 2). No significant difference in the preoperative or postoperative mean STS laxity was seen in patients younger than 15 years compared to patients 15–17 years old (*p* = ns).

**Table 2:** Mean and stratified KT‐1000 arthrometer side‐to‐side (STS) difference values.^a^

	Hyperextension (≤−5°) (*n* = 519)	No hyperextension (>−5°) (*n* = 457)	*p*
Preoperative STS difference, *M* ± SD, mm	3.9 ± 2.7	3.9 ± 2.6	ns
≤2 mm	150 (28.9)	129 (28.2)	ns
3–5 mm	250 (48.2)	206 (45.1)	
>5 mm	119 (22.9)	122 (26.7)	
Patients with available data, *n*	519	457	
Post‐operative STS difference *M* ± SD, mm	2.3 ± 2.3	2.2 ± 2.2	ns
≤2 mm	269 (55.6)	242 (56.5)	ns
3–5 mm	183 (37.8)	157 (36.7)	
>5 mm	32 (6.6)	29 (6.8)	
Patients with available data, *n*	484	428	

*Note*: *M* ± SD: mean ± standard deviation.

Abbreviations: n, number; ns, non‐significant; SD, standard deviation.

^a^ Data are reported as *n* (%) unless otherwise indicated.

Further sub‐analysis showed that the mean preoperative and postoperative STS laxity in patients with passive contralateral knee extension ≤−10° (*n* = 115) did not differ significantly from any of the other subgroups (Table 3) (*p* = ns).

**Table 3:** Mean KT‐1000 arthrometer side‐to‐side (STS) difference values in sub‐analysis of four hyperextension groups.^a^

	Hyperextension (≤ −10°) (*n* = 124)	Hyperextension (−5° to −9°) (*n* = 486)	No hyperextension (−1 to −4°) (*n* = 28)	No hyperextension (≥ 0°) (*n* = 504)	*p*
STS difference^a^ preoperative, *M* ± SD	3.9 ± 2.5	3.8 ± 2.7	4.0 ± 1.7	3.9 ± 2.6	ns
STS difference post‐operative, *M* ± SD	2.3 ± 2.4	2.2 ± 2.2	1.5 ± 1.8	2.2 ± 2.2	ns

*Note*: M ± SD: mean ± standard deviation.

Abbreviations: n, number; ns, non‐significant; SD, standard deviation.

^a^ Data are reported as *n* (%) unless otherwise indicated.

### PROMs

Statistically significant (but non‐clinically relevant) lower scores for the KOOS Sport/Recreation and Quality of Life subscales 2 years post‐operatively were found for the patients in the hyperextension group (≤−5°) (*p* = 0.025 and *p* = 0.049, respectively). No significant differences between the groups were found for the other preoperative or post‐operative KOOS subscales (Table 4) (*p* = ns).

**Table 4:** Preoperative and post‐operative KOOS comparisons.^a^

KOOS	Hyperextension (≤−5°)	No hyperextension (>−5°)	*p*
Symptoms			
Preoperative	591 (76.2 ±17.8)	514 (77.6 ± 17.6)	ns
2 years	237 (80.8 ± 17.5)	212 (83.2 ± 15.7)	ns
Pain			
Preoperative	590 (81.2 ± 16.4)	513 (82.4 ± 16.0)	ns
2 years	238 (88.2 ± 13.4)	212 (90.1 ± 11.3)	ns
ADL			
Preoperative	590 (90.1 ± 14.5)	514 (91.0 ± 13.5)	ns
2 years	238 (94.6 ± 10.8)	212 (96.1 ± 7.4)	ns
Sport/Recr			
Preoperative	568 (56.5 ± 27.1)	493 (60.4 ± 26.9)	ns
2 years	237 (73.2 ± 23.8)	211 (78.6 ± 22.0)	0.025
QoL			
Preoperative	571 (41.0 ± 24.0)	501 (43.9 ± 26.0)	ns
2 years	237 (64.8 ± 25.0)	211 (70.0 ± 22.7)	0.049

Abbreviations: ADLs, activities of daily living; KOOS, knee injury and osteoarthritis outcome score; ns, non‐significant; QOL, quality of life.

^a^ Data are reported as number of patients (mean ± standard deviation). Covariates applied to the model (analysis of covariance) are sex (preoperative and post‐operative KOOS), time from injury to surgery and medial meniscus resection (post‐operative KOOS).

### Revision ACLR

A total of 119 out of 1073 patients (11.1%) underwent revision ACLR within 5 years after primary ACLR. The hazard for revision ACLR in the hyperextension group (11.6%; 65 out of 562 patients) was not significantly different from that in the no hyperextension group (10.6%; 54 out of 511 patients) (hazard ratio, 0.91; 95% CI, 0.63–1.31; *p* = ns).

## DISCUSSION

The most important finding of this study was the lack of association between preoperative physiologic passive KHE (≤−5°) in the contralateral knee and anterior knee laxity, subjective knee function 2 years post‐operatively, or the risk of revision surgery within 5 years after ACLR in patients under 18 years of age using HT autografts in the large majority of cases (93%). Additionally, the study highlighted a notably high overall revision rate, with 11.1% of patients undergoing revision ACLR within 5 years.

Physiologic KHE is common in children and adolescents. In adolescents, normative data of a mean extension of −5° in males (mean age: 14.5 years) and −6° in females (mean age: 14.2 years) have been reported [14]. GJH and KHE are sex‐ and age‐specific, with females of all ages having a higher ability to hyperextend both knees [28], similar to the findings of the present study (68%). The ability to hyperextend the knees decreases from childhood to adolescence [37]. The relatively high prevalence of passive KHE (≤−5°) observed in 53% of the cohort, aligns with previous findings in young patients and exceeds the proportions reported in earlier registry studies involving adults [18, 47].

Kennedy et al. [30] showed already in 1977 that the ACL is maximally taut during hyperextension, and several studies have reported increased force and elongation of the ACL by the end of extension [27, 29, 36]. Furthermore, a correlation between extension capability and impingement pressure of the ACL was registered in a biomechanical study by Jagodzinsky et al. [27]. Biomechanical studies suggest that the ACL becomes taut during hyperextension, which can theoretically be translated into the clinical setting. In this context, KHE may place excessive stress on the ACL graft, potentially increasing knee laxity and the risk of graft failure. However, clinical studies investigating the outcomes after ACLR in patients with KHE have yielded conflicting results. Moreover, most available studies have focused primarily on adult patients, with no dedicated research to date specifically examining outcomes in children and adolescents. In light of the fact that this condition is more common in younger patients, studies in the paediatric population are even more important.

It is difficult to compare the results between previous studies and the present study due to differences in surgical techniques, follow‐up times, rehabilitation protocols, and the different outcomes assessed (e.g., definitions of graft failure). Previous studies in adults have found an association between KHE and graft failure rate, defined as increased post‐operative laxity, with a 5–15 times higher graft failure rate, compared with patients with no hyperextension [20, 23]. Larson et al. [33] found a heel height >5 cm as a predictor of graft failure. The findings of a large registry study by Sundemo et al. [47] suggested that patients with passive KHE had greater anterior tibial translation (measured using a KT‐1000 arthrometer) both preoperatively and 6 months after ACLR with either BPTB or HT autografts. However, when comparing the affected to the contralateral knee, no significant difference in the KT‐1000 STS laxity was observed, neither before nor after surgery [47]. These results indicate that although KHE may be associated with greater anterior tibial translation in the affected knee, it may not necessarily lead to a clinically significant increase in laxity, compared with the contralateral knee. This suggests that KHE may not be as impactful in terms of post‐operative laxity as initially hypothesised, or it may not alter the overall assessment of knee laxity when compared with the healthy knee. The lack of association between KHE and increased anterior knee laxity (STS difference) in the present study may indicate that KHE influences anterior knee laxity differently from what has been suggested in the previously mentioned biomechanical studies. The anatomy of the paediatric knee differs significantly from that of adults. Notably, the development of the femoral intercondylar notch continues throughout skeletal development, with its width decreasing from childhood to adolescence [17]. This suggests that notch impingement, as described by Jagodzinski et al. [27], may be even less relevant in the paediatric population than in adults. Consequently, the biomechanical impact of KHE on the ACL graft, as hypothesised in previous studies on adults, may not be applicable to paediatric patients. Benner et al. [5] found no significant association in adults between KHE and ACL graft failure, measured as a KT‐1000 STS difference >5 mm. A more recent large registry study of 6104 adult patients by Edman et al. [18], used similar outcome measures as in the present study and found no association between KHE (defined as knee extension ≤−5°) and preoperative or post‐operative anterior knee laxity (KT‐1000, STS difference) after ACLR with HT graft. In accordance with the study by Edman et al. [18], the present study found no association between passive KHE and anterior knee laxity in paediatric patients. The threshold of passive KHE used in this study (≤−5°) was derived from adult literature [7, 20]. Helito et al. [23] identified a threshold of 6.5° of KHE where the risk of graft failure (defined by Lachman and anterior drawer test ≥2+, KT‐1000 STS difference >5 mm or pivot‐shift ≥2+ associated with instability complaints) increased significantly. Interestingly, the mean knee extension in the hyperextension group in the present study was 6.1°, which is lower than the threshold identified by Helito et al. [23]. This might have led to an underestimation of the impact of KHE on clinical outcomes. To better assess the paediatric population, we conducted a subgroup analysis with four KHE categories, which revealed no significant association between the post‐operative KT‐1000 STS difference and KHE subgroups (Table 3).

Helito et al. [23] and Guimarães et al. [20] found inferior post‐operative subjective knee function (IKDC and Lysholm score) in adult patients with KHE. Similarly, Kim et al. [31] found lower Lysholm and IKDC scores in patients with KHE, and Larson et al. [33] also reported poorer subjective knee function (IKDC, Cincinnati knee ratings scale and Lysholm score) in patients with KHE. On the contrary, Benner et al. [5] found no association between KHE and subjective knee function (IKDC and Cincinnati knee ratings scale). Similarly, Edman et al. [18] found no clinically significant differences in preoperative or 1‐, 2‐ or 5‐year post‐operative KOOS subscale scores between patients with KHE and patients with no KHE. The present study found a statistically significant difference between the hyperextension and no hyperextension groups in the KOOS Sport/Recreation and Quality of Life subscales at the 2‐year follow‐up. However, these differences were less than the suggested minimal important change (MIC) for the KOOS (8–10 points) and were hence non‐clinically relevant [2, 42, 44]. Further, no differences were seen in the remaining KOOS subscales.

The literature regarding KHE and its association with graft failure or revision surgery after ACLR in adult patients is conflicting, and there are difficulties in comparing the results due to the different definitions of graft failure, such as graft rupture or revision ACLR. Edman et al. [18] found no difference in the rate of revision surgery within 5 years after ACLR using HT autografts between patients with KHE (≤−5°) and those without hyperextension (>−5°). In a recent study, Cristiani et al. [9] found no association between KHE (≤−5°) and revision surgery within 5 years after ACLR reconstruction in patients younger than 20 years. Similarly, no association between passive KHE (≤−5°) and revision surgery ≤5 years after ACLR was found in the present study, where 93.3% of the primary ACLRs were performed with HT autografts. On the basis of the results of our study, passive KHE alone should not be considered as a contraindication for the use of HT grafts in the paediatric population. The rate of revision ACLR in children and adolescents has been reported to be higher than in adults in several studies, with revision rates ranging from 3% to 19% [1, 8, 19, 21, 35, 45]. Edman et al. [18] reported an overall revision rate in adults of 4.9% within 5 years after primary ACLR [18]. The finding of a revision rate of 11% in this study aligns with previous results in the paediatric population.

The findings of this study have important clinical implications. Paediatric patients with KHE can expect similar outcomes in terms of anterior knee laxity, subjective knee function, and revision ACLR rates to those without KHE after ACLR, but paediatric patients may face a higher risk of revision surgery following ACLR when compared to adults.

The major strengths of this study were the large number of paediatric patients (*n* = 1250) as well as the long‐term follow‐up after revision surgery (5 years). Further, the occurrence of revision ACLR was identified in the SNKLR, which covers the whole nation. Another strength was that all patients underwent the index surgery using a standardised technique at the same facility.

This study has several limitations. First, inter‐observer variability may exist in the ROM and arthrometric laxity measurements. However, all physiotherapists who performed the measurements were experienced in assessing passive knee ROM using a goniometer and laxity with the KT‐1000 arthrometer. In addition, the technique of ROM and laxity assessment was standardised. Second, the cutoff for defining KHE in the paediatric population is unknown. In this study, a cutoff of −5° was used based on previous studies in adult patients [7, 18, 20, 33]. Third, only anterior knee laxity was measured. The association between passive KHE and rotatory laxity was not assessed as the pivot‐shift test was not registered in a standardised manner in our database. Fourth, there was no uniformity in assessing post‐operative passive knee joint laxity in paediatric patients after ACLR [52]. However, the KT‐1000 arthrometer is the most commonly used method [52], and it has been validated in children with ACL injuries [16]. The subjective outcome used in our study was the adult version of the KOOS instead of the suggested paediatric version. The original KOOS has been shown to be hard to understand for children and misinterpretation may occur, which is a limitation and contributes to the difficulty in interpretation of the results [40]. Fifth, no available information about post‐operative activity levels was available in our database, and this potential confounder could therefore not be analysed in the revision ACLR analysis. However, the pre‐injury Tegner activity levels did not differ between the two groups, and this could eventually give an indication of a similar distribution of post‐surgical Tegner activity levels between the groups. Sixth, we had no information on graft rupture, as only revision ACLR was registered in the SNKLR. Therefore, possible differences in the graft rupture rates between the groups could not be ruled out. Finally, 6.7% of all patients included in this study underwent ACLR with BPTB graft, and analysis of this subgroup alone was not possible due to lack of statistical power. Hence, the results of this study are only applicable to HT grafts.

## CONCLUSION

There was no significant association between preoperative passive contralateral KHE and anterior knee laxity, subjective knee function or the risk of revision ACL surgery in paediatric patients. These findings suggest that KHE alone should not preclude the use of HT grafts in children and adolescents undergoing ACLR. The study found a high rate of revision ACL surgery in this paediatric population.

## AUTHOR CONTRIBUTIONS

Conceptualisation, data processing, statistical analysis, methodology and manuscript writing: Frida Hansson. Methodology and critical review of the manuscript: Anders Stålman, Per‐Mats Janarv and Eva Bengtsson Moström. Statistical analysis and critical review of the manuscript: Gunnar Edman. Conceptualisation, methodology and critical review of the manuscript: Riccardo Cristiani. All authors have given final approval for the version to be published.

## CONFLICT OF INTEREST STATEMENT

The authors declare no conflicts of interest.

## ETHICS STATEMENT

Ethical permission for this study was obtained from the Regional Ethics Committee of Karolinska Institute (Diarie number: 2016/1613‐31/2).

## Data Availability

Data are available from the corresponding author F. H. on request.
